# Assessing Occupational Work-Related Stress and Anxiety of Healthcare Staff During COVID-19 Using Fuzzy Natural Language-Based Association Rule Mining

**DOI:** 10.3390/healthcare13141745

**Published:** 2025-07-18

**Authors:** Abdulaziz S. Alkabaa, Osman Taylan, Hanan S. Alqabbaa, Bulent Guloglu

**Affiliations:** 1 Department of Industrial Engineering, Faculty of Engineering, King Abdulaziz University, Jeddah 21589, Saudi Arabia; 2 Department of Management Engineering, Faculty of Management, Istanbul Technical University, 34367 Istanbul, Türkiye; 3 University Medical Services Center, King Abdulaziz University, Jeddah 21589, Saudi Arabia; halkabaa@kau.edu.sa; 4 Department of Economics, Faculty of Management, Istanbul Technical University, 34367 Istanbul, Türkiye; guloglub@itu.edu.tr

**Keywords:** mental health, occupational stress, burnout, resilience, fuzzy association rule mining, depression

## Abstract

**Background/Objective:** Frontline healthcare staff who contend diseases and mitigate their transmission were repeatedly exposed to high-risk conditions during the COVID-19 pandemic. They were at risk of mental health issues, in particular, psychological stress, depression, anxiety, financial stress, and/or burnout. This study aimed to investigate and evaluate the occupational stress of medical doctors, nurses, pharmacists, physiotherapists, and other hospital support crew during the COVID-19 pandemic in Saudi Arabia. **Methods:** We collected both qualitative and quantitative data from a survey given to public and private hospitals using methods like correspondence analysis, cluster analysis, and structural equation models to investigate the work-related stress (WRS) and anxiety of the staff. Since health-related factors are unclear and uncertain, a fuzzy association rule mining (FARM) method was created to address these problems and find out the levels of work-related stress (WRS) and anxiety. The statistical results and K-means clustering method were used to find the best number of fuzzy rules and the level of fuzziness in clusters to create the FARM approach and to predict the work-related stress and anxiety of healthcare staff. This innovative approach allows for a more nuanced appraisal of the factors contributing to work-related stress and anxiety, ultimately enabling healthcare organizations to implement targeted interventions. By leveraging these insights, management can foster a healthier work environment that supports staff well-being and enhances overall productivity. This study also aimed to identify the relevant health factors that are the root causes of work-related stress and anxiety to facilitate better preparation and motivation of the staff for reorganizing resources and equipment. **Results:** The results and findings show that when the financial burden (FIN) of healthcare staff increased, WRS and anxiety increased. Similarly, a rise in psychological stress caused an increase in WRS and anxiety. The psychological impact (PCG) ratio and financial impact (FIN) were the most influential factors for the staff’s anxiety. The FARM results and findings revealed that improving the financial situation of healthcare staff alone was not sufficient during the COVID-19 pandemic. **Conclusions:** This study found that while the impact of PCG was significant, its combined effect with FIN was more influential on staff’s work-related stress and anxiety. This difference was due to the mutual effects of PCG and FIN on the staff’s motivation. The findings will help healthcare managers make decisions to reduce or eliminate the WRS and anxiety experienced by healthcare staff in the future.

## 1. Introduction

The COVID-19 virus, also known as SARS-CoV-2 in the literature, was declared a pandemic by the World Health Organization [1]. The investigations showed that the health staff faced serious problems while interacting with crowds in hospitals, and intensive working environments [2] were the reason for depression, anxiety, and burnout [3]. Sources of WRS and anxiety arise from work, organization, and occupation-related problems caused by the individuals, management, and environment [3]. The sources of WRS and anxiety are diverse and arise from the social role of employees, work-related uncertainties, conflicts, and increased workload. For instance, long working hours, high workload, unemployment, a lack of community support, and an uncomfortable physical environment are the main depression risk factors and can be considered as the signs of anxiety among public employees, especially women [4]. An empirical assessment of job stress and job performance involving 204 participants was carried out, as presented in [5]. The multiple reasons for the demotivation and low productivity of healthcare workers are various and include the lack of adequate equipment for treatment and the emergence of new diseases. As a result, all demotivating factors cause physical and mental fatigue and negatively affect human relations, create an imbalance between work and personal responsibilities, cause anxiety, increase difficulties, reduce the reputation of organizations, and change the working climate [6]. Numerous studies in the literature indicate that WRS factors negatively impact employee satisfaction and performance [7] while also increasing their workload and affecting their colleagues [8]. In this study, the factors arising from WRS during COVID-19 and their effect on healthcare staff performance were investigated. The dependent and independent factors (causes of diseases) were identified by a thorough review of the existing literature and interviews with the key staff of hospitals. Consequently, it was determined that the most significant sources of work stress and anxiety arising from COVID-19 were primarily rooted in financial and psychological challenges, along with socio-demographic factors of the staff. Moreover, it was also determined that associations (functional relations) between the dependent and independent factors of the disease and their complex dynamics have impacts on the healthcare system and staff, as with factors like anxiety and burnout.

Anxiety can lead to diseases and occupational injuries which adversely influence the physical, social, and psychological health of the healthcare staff. However, burnout is the psychological reaction of healthcare staff, and both of these cases have a significant relationship. Anxiety can trigger burnout syndrome, including psychological and behavioral actions due to work stress and pressure on healthcare staff who are exposed to burnout due to the nature of the work area. Anxiety and burnout undoubtedly carry the risk of causing a decrease in the functionality of healthcare professionals. WRS factors can be identified by numerical (crisp) and non-numerical (linguistic) information [9,10,11]. Anxiety, burnout, and work-related stress are qualitative factors (fuzzy linguistic variables) and can more accurately be defined by fuzzy linguistic terms such as ‘*very low*’, ‘*low*’, ‘*medium*’, ‘*high*’, or ‘*very high*’. Qualitative knowledge is described by the ambiguous and imprecise ordinary linguistic terms that are obtained from domain experts who are familiar with the target health system. On the contrary, numerical information can be obtained from several measurable sources [12]. One important AI technique, fuzzy sets and systems, can be employed to handle qualitative knowledge defined on a restricted vague domain whose explanation is not possible by numerals [13,14]. Healthcare systems are complex and need fuzzy sets and systems to associate fuzzy linguistic variables with their term sets [12,15] and fuzzy rules. Hence, a range of fuzzy mathematical-based rules may help to formalize and develop a fuzzy model for an ill-defined complex healthcare system. The fuzzy rule-based systems can be represented by fuzzy association rule mining (FARM) approaches. FARM uses fuzzy set theory and considers linguistic variables for building explainable reasoning systems, and it is a well-known data mining method to identify repeatedly occurring patterns from datasets. FARM has a variety of applications in numerous fields, such as customer segmentation [16,17], fraud detection [18,19,20], market basket analysis, and demand choices [21,22]. In the era of big data, to uncover the hidden relationships between health factors, health organizations have to solve the issues of overlapping data and interpret them. The absence of clarity regarding health-related factors leads to vagueness, ambiguity, and imprecision. Therefore, Boolean association rules are not suitable for solving the overlapping and boundary problems of these data points [21] and decide on the optimal number of fuzzy rules. For instance, one negative health factor may be the cause of more than one disease; hence, a data point related to a disease can be the indication of many diseases, and we can consider this issue as being the member of many clusters in fuzzy logic. A fuzzy inference system (FIS) uses fuzzy logic to carry out the following: determining fuzzy linguistic variables and their linguistic term sets, membership functions, fuzzy rule base, fuzzification process, compositional rule of inference (inference engine), and defuzzification of the findings. The application areas of FIS are various and include control systems, decision-making processes, and pattern recognition. FARM is a rule mining approach to identify operations of linguistic variables and terms to establish rules using natural language. The FARM approach can produce more dependable and accurate solutions even if the health data overlap and are positioned in neighboring boundaries. Since traditional approaches cannot consider problems of multiple clustered data, fuzzy set theory can enhance the association of factors using the FARM method [21,23,24] and solve the overlapping issues for data cases related to work stress problems. Similarly, the cognition science and cognitive architecture presented in [25] stated that people base their thinking principally on mental images and use vague and imprecise natural conceptual patterns rather than numerical (crisp) quantities to communicate. Since fuzzy sets can help data mining to discover strong recurrent patterns recognized widely for fuzzy associations, FARM can identify operations of linguistic variables and terms to establish rules [26] using natural language. For example, the rule ‘*If health workers are experiencing socio-demographic changes (SCF)*, *they are likely to suffer psychological (PCG) and financial (FIN) issues*’ is an association rule that can be mined from the dataset using simple fuzzy linguistic terms. Such rules can be used to reschedule hospital staff’s working hours in order to improve living standards and the quality of the workplace environment. FARM can also employ rules to describe the relations between different items in the dataset and enable researchers to reveal hidden patterns in large datasets [27]. The fuzzy clustering algorithms presented in [28,29] provide more suitable partitions of datasets for optimizing the number of fuzzy rules in a fuzzy logic model. In this approach, each group of a dataset is represented by a fuzzy rule, so the data point close to the group’s center will have a higher membership degree and become the part of that cluster [30]. A study given in [31] justified the relevance of fuzzy logic applied to FARM in their data mining setup. The great advantage of fuzzy clustering algorithms is that each data point belongs to a cluster with a degree of membership between [0, 1] for representing the data and its membership degree [32].

**Figure 1:**
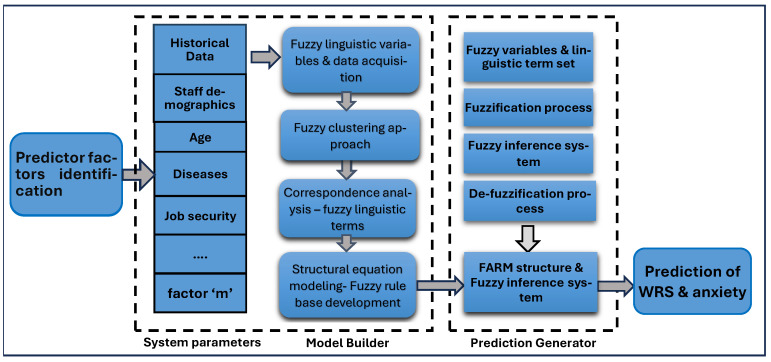
Fuzzy association rule mining model for WRS and anxiety prediction.

The objective of this study was to conduct a survey with doctors, nurses, dentists, psychotherapists, health technicians, and medical secretaries working in public and private medical organizations in Saudi Arabia to disclose WRS and anxiety. The data obtained from the survey were used to develop a FARM method for determining frequently repeated issues of WRS experienced by health staff. This study also aims to determine the relevant health factors that are the root causes of WRS and anxiety for better preparation and motivation of staff in reorganizing resources and equipment in hospitals properly to enhance healthcare services. The outcome of this study will support decision makers of the healthcare sector to determine what kind of parameters should be closely considered in hospitals to increase the performance of staff. It also offers a new methodology to extend traditional association rule mining with fuzzy set theory and fuzzy clustering algorithms in addition to statistical approaches such as structural equation modeling, correspondence analysis, analysis of variance (ANOVA), and analysis of WRS. Additionally, FARM was used to identify the most significant factors among the eleven variables, including staff demographics, age, body mass index, pre-existing conditions, job security, sources of infection, symptoms before hospital admission, radiographic signs of pneumonia, length of hospital stay, patient discharge, and current incidence rates for WRS assessment. Figure 1 shows the FARM model for WRS and anxiety prediction. Data were collected from health organizations’ staff with the following demographics: age between 18 to 60, both male/female, single/married, and Saudi/non-Saudi. The health organizations investigated are leaders in the sector, with over 50 thousand visitors per month from different cities in the Kingdom.

Hence, Section 1 introduces the WRS problems, anxiety, and burnout factors, as well as the fuzzy association rule mining (FARM) method and the factors of healthcare issues. Section 2 presents a comprehensive literature review of studies on COVID-19 and the impact of chronic WRS and anxiety on both employees and organizations. Section 3 includes the methodology to present the FARM, statistical analysis of the consequences of COVID-19 outbreak, financial consequences, clustering analyze of WRS and anxiety, correspondence analysis, and the structural equation modeling approach. In Section 4, the results and findings of this study are presented, the FARM approach is detailed, and the findings are analyzed. In Section 4.1, fuzzy natural language-based association rule mining of WRS and anxiety is synthesized. Section 5 gives the conclusions, in which the qualitative and quantitative data obtained from a survey of healthcare sector employees are analyzed, and their consequences are presented.

## 2. Literature Review

COVID-19 globally affected the psychology of people, including healthcare workers. Stress, depression, and anxiety were the most common impacts that affected these societies. In the early period of COVID-19, the WRS and anxiety of healthcare workers were not taken into serious consideration. However, in its later periods, when mass death tools appeared in some countries, and the insufficiency of staff became apparent, the dimensions and the consequences of the COVID-19 issue were better understood. Then, physiological and sociological factors affecting the healthcare workers became much more important. Additionally, it was important to examine all factors that caused stress and anxiety, their mutual interractions, and their effects on the healthcare staff. Therefore, a fuzzy approach for time series forecasting of COVID-19 was carried out by Alyahya & Abo Gazalah [7], considering several job stress factors to discover the presence of depressive symptoms. Chung et al. developed a scale for assessing WRS an anxiety in healthcare staff in response to viral epidemics [1]. Nasri et al. [33] developed a novel fuzzy neural network time series prediction model for investigating the relationship between clinical factors of the viral epidemic stress of government employees, their unhappiness, and anxiety. Tính & Điều [34] developed a hybrid model of fuzzy C-means and SVM for combining health and behavioral science to reduce healthcare staff burnout due to COVID-19. The key recommendation in the study was to meet the challenges posed by the pandemic which drove a necessary cultural change and improved public health systems.

Sharif et al. [35] computed a tomography scan in COVID-19 for meta-analysis and measuring the anxiety of healthcare personnel working in two Finnish private medical care centers to determine mental health resilience of nurses and anxiety using demographic variables. Egrioglu et al. [36] investigated the effects of WRS and anxiety on job satisfaction, self-esteem, organizational trust, and commitment in tourism and accommodation businesses. Mohammed et al. [37] aimed to explore several potential determinants of job satisfaction by working from home and maintaining work-life balance, and they found that WRS has both direct and indirect significant effects on job satisfaction.

Fatima & Pasha [38] examined the connection between WRS and COVID-19 using Machine Learning Techniques in Medical Diagnosis. Ampavathi and Saradhi [39] employed linear and logistic regression models to study antecedent, demographic, and professional factors on the health impairment, motivation, and organizational outcomes of nurses. Lu et al. [6] evaluated the relationship between stressors and stress levels using sociodemographic data, conflicts and uncertainty in occupational factors, social support, and the total level of stress experienced. D’emeh et al. [40] identified an expanded nursing stress scale (ENSS) that included quality of life during the COVID-19 pandemic and found that gender, job type, education, and dealing with infected patients were important factors. Widhiastuti et al. [41] analyzed the impact of work stress on job performance during COVID-19 and studied the effects of job burnout considering several factors. Zareei et al. [42] studied burnout among nurses during the COVID-19 pandemic working in highly contagious environments and found that employing assistant nursing showed significant differences in work stress, willingness to work, and patient satisfaction scores. Professions that require human-to-human interaction, such as nursing, teaching, psychology, and medicine, have been examined to determine the impact of chronic WRS and anxiety on both employees and organizations and presented in [43,44,45,46,47]. On the other hand, the studies for determining the association between WRS and remote work suggested exciting visions to motivate staff quickly. This is because it contains less stress and overload on family matters [48], impact on physical and career performance [49], and individual work stress time [50]. The studies given in [48,51,52,53] aimed to build a theory to determine the “over-loading role” as well as the “spreading effect” of experiencing the pandemic at home and during work for revealing the greater stressors for women directly stemming from job overload [48]; working in hazardous occupations in lower socio-economic classes [52,54]; and in cases where the support of family, spouses/colleagues, and institutions are limited and conflicted [53]. Anger et al. [52] provided a comprehensive assessment by screening 5158 science journal articles and found 118 different interventions that had been offered to healthcare workers. They focused on the support and treatment of mental health and collected valuable information about methods to improve mental health among healthcare workers. Norhayati et al. [55] determined the estimated prevalence of the psychological impacts of COVID-19 among healthcare providers in the Asian region and found high prevalences of stress, depression, anxiety, and insomnia, but low prevalences of post-traumatic stress disorder, among healthcare providers. Paschalia et al. [56] used descriptive statistics and chi-square approaches for data analysis to identify relationships between WRS levels and quality of life for health workers during the COVID-19 pandemic. They used questionnaires and stress scales to collect data. Anger et al. [52] addressed the mental health of healthcare workers by a systematic review of evidence-based interventions and revealed that there was a significant relationship between WRS and quality of life. They found out that WRS is a feeling of pressure experienced by employees in dealing with work and has the symptoms such as feeling uneasy, feeling alone, unstable emotions, smoking excessively, being anxious, nervousness, and suffering from high blood pressure. To examine the correlations between the psychological stressors, coping strategies, and emotional responses of healthcare workers during the COVID-19 pandemic, Puia et al. [57] examined differences among demographic and occupational groups through a cross-sectional survey conducted among 338 healthcare workers, including physicians and nurses, in urban and rural healthcare settings during the pandemic. They used statistical analyses and included descriptive, inferential, and correlation techniques to assess relationships between variables. Le et al. [45] evaluated the psychological effects of COVID-19 on healthcare workers in Vietnam to determine the effects of social distancing. Master et al. [46] investigated the psychological impact of the COVID-19 outbreak on frontline nurses using multiple logistic analysis to reveal the concern for family, being treated differently, and negative coping styles, and their study revealed that stress symptoms were found to be positively related to psychological distress. Kroes et al. [44] investigated workforce perspectives of a harm reduction facility in a residential mental healthcare setting and found that staff had increased knowledge and confidence in addressing harm reduction issues with consumers, suggesting that interactions between staff and service users were enhanced.

Morán et al. [47] carried out a systematic review regarding the consequences ofWRS due to the COVID-19 pandemic to address several parameters such as work stress in health workers, in remote workers, in agricultural workers, in restaurant workers, in teaching workers, and in prison workers for determing their depression and anxiety levels. The results obviously showed that the healthcare staff experienced the greatest work stress. Sahebi et al. [54] used random effects for meta-analyses to carry out an umbreall review to determine the prevalence of depression and anxiety among healthcare staffs during the COVID-19 pandemic. Sari & Khaira [58] investigated the impacts of the COVID-19 pandemic lockdown on university students’ lives, which often led to higher levels of stress.

## 3. Material and Methods

### 3.1. Data Collection Method

A cross-sectional survey was conducted among 204 healthcare staff workers, including nurses (41), medical doctors (21), health technicians (19), medical secretary (16), dentists (44), physiotherapists (12), pharmacists (18), engineers (11), and other employees (servants) (22). Validated instruments were used for data collection about the factors as decsribed in the abstract and introduction. Primary data were collected through an online survey designed as a Google form, sent via email randomly to 150 hospital staff in remote areas. Over the course of their study, responses from 96 staff were received online. In addition, the remaining 108 survey results were obtained from hospital staff in nearby areas by randomly distributing the surveys directly to healthcare personnel.

Questions were in both Arabic and English to enable the respondents to choose a language of their own. The survey questions were designed, checked, and validated after reviewing several similar studies, consulting different authors, a family doctor, and a government doctor, who helped make some changes to the questionnaires. The survey was divided into four categories: (1) questions designed to reveal socio-demographic characteristics, including gender, age, marital status, occupation, the number of people living in the household, the number of hours spent outside their homes before the quarantine, and their opinions about the lockdown; (2) questions aimed to reveal anxiety; (3) questions aimed to determine the financial impact of COVID-19 on the staff; and (4) questions intended to determine the staff’s psychological stress during the COVID-19 pandemic. Then, the obtained dataset was analyzed by several techniques, such as correspondence analysis, the structural equation model, and the fuzzy association rule mining (FARM) approach, to predict the WRS and anxiety of healthcare staff. Qualitative metrics and quantitative statistics were employed to develop a FARM approach to reveal WRS and anxiety factors. Considering all the abovementioned methods for developing the FARM approach, nine fuzzy rules were constructed for association rule mining to determine the leading parameters.

Validity and formal difficulties have arisen in the acquisition of medical information, especially due to ethical issues regarding the rights of healthcare staff. This study was generally a non-interventional cross-sectional study, which did not allow us to scientifically or medically correlate causes and effects between the observed phenomena and directly take into account the privacy of participants or patients. Participants were all informed about the purpose of the study and the protection of their personal data and privacy. The participants were from different work specialties, which means a specific class of staff was not investigated; also, the findings did not have a feature that directly targeted the individuals or a purpose that might cause them distress and/or reveal their personal privacy. In addition, we conducted this research in accordance with the Declaration of Helsinki and received approval from the Unit of Biomedical Ethics, Research Ethics Committee (REC), King Abdulaziz University, with NCBE Registration No: (HA-02-J-008) on 24 March 2025.

### 3.2. Fuzzy Association Rules Mining (FARM) and Fuzzy Transactions

This section mainly covers the scope of this study, the questionnaire, and the sample design used for data collection, the obtained results, and the rule association. The qualitative data contain nominal or ordinal data and have finite non-ordered values demonstrated by gender data (male/female), whereas ordinal data, like fuzzy sets, incorporate finite ordered values. Hence, customer credit ratings, health care services, etc., can be illustrated and identified by fuzzy linguistic terms such as ‘bad, fair, and excellent.’ Depending on the professional requirement, the data mining task can be of a relational or predictive (regression or classification) type. Thus, FARM can be successfully used to discover relationships between diseases, symptoms, and diagnosis and patients’ characteristics, treatments, genes, and functional relations. A collection of the terms forms the phrases of the natural language called linguistic rules (composite terms) to associate the parameters. Extensive deep psychological problems, destructive financial problems, socio-demographic changes, etc., are examples of composite terms. Cognitive patterns used by humans and mental images are apparently quite vague and can best be represented by linguistic variables in fuzzy sets [25,59]. A membership function (MF) $\mu_{\tilde{M}}\left(\alpha ,y\right)$ of a fuzzy set $\tilde{A}\;$ portrays the fuzziness of variables to map the linguistic terms and their interpretation, as seen in Equation (1).

(1) $$\mu_{\tilde{M}}\left(\alpha ,y\right)=\mu_{\tilde{A}}(y)$$

Here, α is a fuzzy linguistic variable and its value are defined by *μ_α_*(*y*) and interpreted in linguistic terms. A number of linguistic terms can be combined with connectors such as ‘*and*’, ‘*or*’, and ‘*not*’. If α and β are two atomic terms (WRS, anxiety, occupational health, age, etc.) in a universe of discourse U (diseases related to WRS), then a composite rule can be defined in universe Y, as given in Equation (2).

(2) $$\alpha \;or\;\beta :\mu_{\alpha \;or\;\beta }\left(y\right)=\max\left(\mu_{\alpha }\left(y\right),\;\mu_{\beta }\left(y\right)\right),\;$$

$$\alpha \;and\;\beta :\mu_{\alpha \;and\;\beta }\left(y\right)=\min\left(\mu_{\alpha }\left(y\right),\;\mu_{\beta }\left(y\right)\right),$$

$$Not\;\alpha =\bar{\alpha }:\mu_{\bar{\alpha }}\left(y\right)=1-\mu_{\alpha }\left(y\right).$$

FARM is a data mining and machine learning approach for associations of ‘*If-Then*’ rules and shows the attribute-valued conditions in a dataset [60]. ‘*If-Then*’ rules are well-stated relations of linguistic variables and their term sets. The non-linear relationships found in ill-defined systems are articulated through fuzzy relational equations. These relations can be represented using a range of fuzzy compositional operations applied to membership functions (MFs) for defining overlapping partitions of the antecedent and consequent spaces of attributes. Since a dataset containing several data records comprises a set of attributes, it can be symbolized by *A→B* to show the fuzzy relations. This relation is called a fuzzy rule, and its structure is ‘*If*’ (shown by *A*), the consequent is ‘*Then*’ (shown by *B*). Fuzzy rules hypothetically mean that if ‘*A*’ occurs in the dataset, then consequent ‘*B*’ also occurs. For instance, ‘*If health workers are likely experiencing socio-demographic changes (A)*, *Then they may suffer psychological and financial problems and may suffer high WRS and anxiety (B)*’.

Multivariate analysis contributed to association rule mining (ARM) for better understanding and discovery of interesting correlations among factors [9]. The bottleneck of this technique is its inability to directly mine quantitative attributes. In a dataset with ‘*m*’ attributes and ‘*n*’ values, ‘*n − 1*’ antecedents (*A*) and one consequent (*B*) can be generated, which represent the maximum of ‘*nm^n − 1^ −1*’ rules. Therefore, FARM can generate many rules from a given quantitative dataset that has a hidden relationship, and its rules are more informative than rules with crisp values. However, not all rules are meaningful [61], and the statistical significance and relationship of antecedent and consequent components of fuzzy rules can be evaluated by chi-square testing. The partitions of fuzzy rule pairs of ‘*A*’ and ‘*B*’, and the support value of a rule set referring to the data recorded, in either set ‘*A* or *B*’ are used to eliminate redundant rules. Three measures, support, confidence, and lift, are used for the assessment of fuzzy rules. Hence, a set ‘*A*’ including data points (x) in a universe U and has a support value such that $\mu_{A}\left(x\right)>0:$

$$Support(A)\;=\;\left\{x|\mu_{A}(x)>0\right\}.$$

The support of a fuzzy set ‘*A*’ is the set of all points *x ∈ U* such that *μ_A_(x) > 0*, which is defined as

$$F-Supp(\underline{A})=\{x|\mu_{\underline{A}}(x)>0,\;x\;\in \;U\}.$$

F-Supp (Fuzzy support) values of a fuzzy set are computed by Equation (3).

(3) $$F-Supp\left(A\to B,\;\tau \right)=\frac{\sum_{k=1}^{N}min\left\{t_{k}(z_{i})\right\}}{N}$$

A FARM ‘*A→B*’ holds if and only if $\tilde{\tau }(A)\leq \tilde{\tau }(B)$ for each $\tilde{\tau }\in T,$ which means that for every fuzzy transaction ($\tilde{\tau }),$ the insertion degree of ‘*B*’ is greater than that of ‘*A*’ in a fuzzy set. Hence, the confidence of a fuzzy rule is computed as follows:

(4) $$F-Conf\left(A\to B,\;\tau \right)=\frac{F-supp(A\cup B)}{F-supp(A)}$$

Considering all this, the support and confidence values refer to the percentage of transactions and the estimate of the conditional probability of the outcome of the antecedents, respectively [62].

Since the association between *psychological and financial impacts* is vague and non-linear, these associations usually include complex forms of knowledge described by fuzzy linguistic terms and rules. The fuzzy linguistic variables and terms sets are presented in Section 4.5 of the Results and Findings section. The stress level identification and the impact of factors on medical staff’s WRS and anxiety are illustrated in Section 4.5. For instance, suppose that the patients’ demographic distribution, age, and mass index were checked to determine the underlying diseases, job satisfaction, infection source, and symptoms of depression and anxiety; the statements ‘*old age*’, ‘*high body mass index*’, and ‘*high anxiety disorder*’ are called the antecedents of the fuzzy linguistic variables to define the linguistic terms naturally. The detailed application of theFARM approach can be found in Section 4.5.

## 4. Results and Findings

### 4.1. Statistical Analysis and Consequences of COVID-19 Pandemic

**Figure 2:**
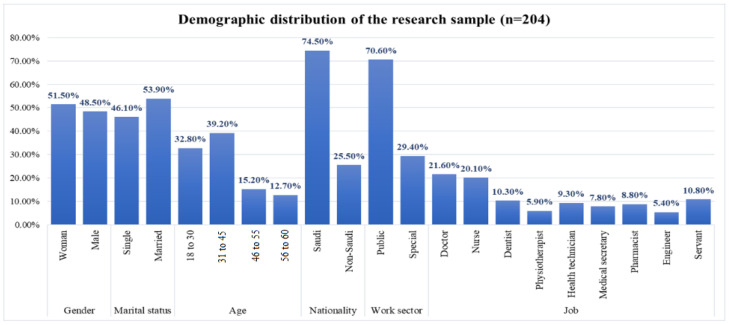
Distribution of research samples.

Our survey consisted of 40 questions; the first 13 questions were about socio-demographic factors. The questions ordered from 14 to 40 were about the technological, psychological, and financial impacts of WRS and anxiety levels during and after the COVID-19 pandemic. Figure 2 shows the distribution of gender, marital status, age group, nationality, working sector, and occupation according to the categories of demographic data. The results of statistical analysis presented in the following sections will be used to establish a fuzzy logic based approach that can be employed for developing a fuzzy association rule mining structure and to predict WRS and anxiety.

#### 4.1.1. Psychological Consequences

**Figure 3:**
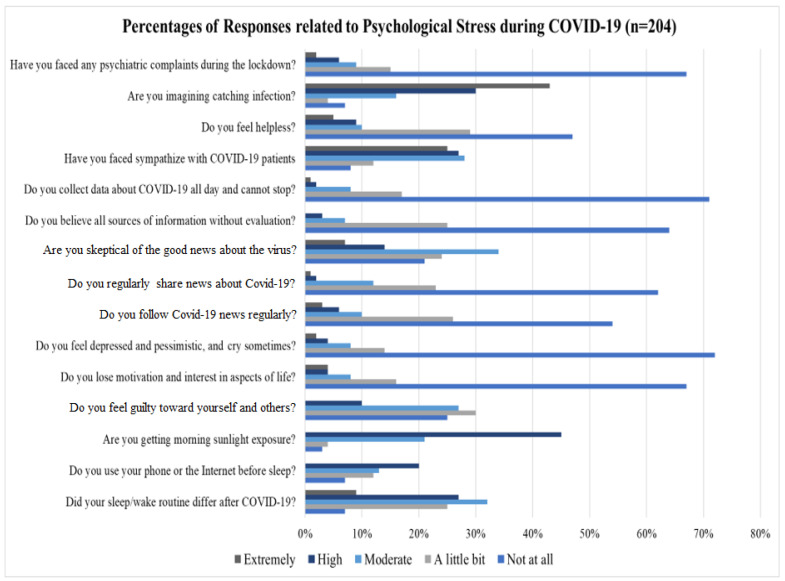
Percent of healthcare staff suffered psychological stress during COVID-19 pandemic.

The emotional distress of healthcare staff experienced during the COVID-19 pandemic was measured using a 5-point Likert scale ranging from 0 (Not at All) to 4 (Extremely). As shown in Figure 3, the responses related to psychological stress presented that 68% of the staff did not have any psychiatric complaints, only 8% of them reported high and extremely high psychiatric complaints, and about 43% of the respondents had concerns about getting the virus while performing their job. In contrast, about 11% believed the likelihood of contracting infection during missions was negligible or very low.

On the other hand, the detailed analysis presented in Figure 3 showed that most of the staff did not feel helpless during outbreaks, they shared feelings of sympathy toward the COVID-19 patients, only about 14% stated that they felt highly or extremely helpless. About 79% of staff conveyed their lack of skepticism regarding positive news about the virus, while 21% of them were very skeptical. Interestingly, 85% of healthcare staff confessed that they did not share COVID-19 news. Additionally, a significant number of healthcare staff showed strong resilience to psychological stress, since 72% responded with ‘*not at all*’, and 14% replied ‘*a little bit*’ to the queries about feelings of depression and pessimism. Furthermore, a substantial majority of staff affirmed that they never lost motivation and considered health service as a part of their profession. In fact, emotional issues that have psychological consequences cannot be adequately explained and described by numerical (crisp) value alone. They are called fuzzy linguistic factors in fuzzy logic and can be represented better by fuzzy linguistic terms such as ‘his emotional situation is very bad, or extremely depressing, or she is emotionally very strong, etc.’ These are all the subjects of fuzzy logic.

#### 4.1.2. Financial Consequences

**Figure 4:**
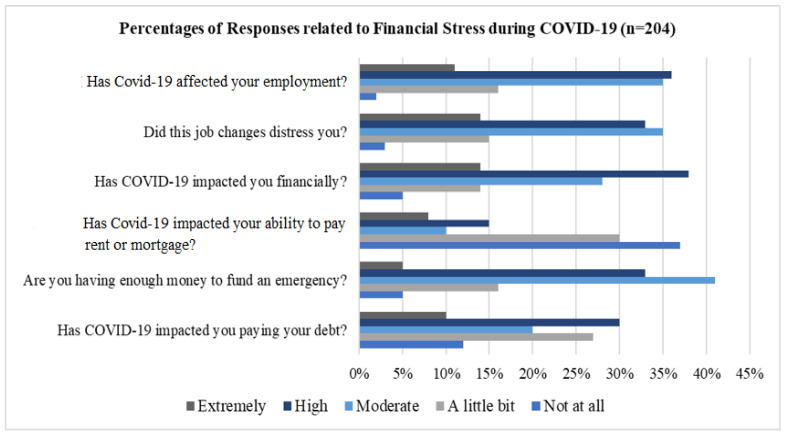
Percent of staff suffered financial stress during COVID-19 pandemic.

The financial challenges that the staff encountered during COVID-19 are illustrated in Figure 4, which demonstrates that the outbreak had a significant influence on their employment. Over 15% and 8% expressed that their capacity to pay their mortgage or rent was highly and extremely affected by the COVID-19 pandemic, respectively. While 40% of respondents reported being moderately affected, 33% of healthcare workers reported having sufficient resources to finance emergencies. Finally, 30% of healthcare workers asserted that the COVID-19 pandemic had a substantial impact on their ability to pay their debts. These findings confirm that the COVID-19 pandemic had a noteworthy impact on the financial well-being of staff when compared with the financial stress, which showed relatively less disparity than psychological stress. Although finance is a measurable parameter, financial stress is not; it is a qualitative parameter and can be much better explained with fuzzy linguistic terms and antecedents like ‘*The financial stress is very high*’, ‘*The psychological (PCG) impact is very low in this organization*’, ‘*There is no financial issue at all*’, etc.

#### 4.1.3. Work-Related Stress and Anxiety Consequences

**Figure 5:**
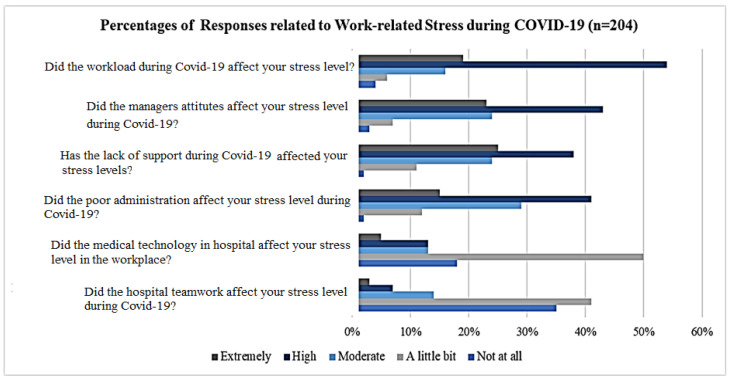
Distribution of WRS during COVID-19 pandemic.

About 73% of staff reported that their workload affected their WRS. As seen in Figure 5, poor management emerged as a primary contributing factor for WRS, resulting in 43% of healthcare staff suffering from high stress, with 23% strongly agreeing with this statement. More than 60% of healthcare staff believed their stress at work was due to a lack of support during the pandemic. Conversely, the utilization of technology within the hospital was not identified as a stress-inducing factor. More than 70% of healthcare specialists affirmed that teamwork did not contribute to WRS negatively. Briefly, the findings illustrated that high workload, managerial shortcomings, and insufficient support yielded the highest WRS, and they are the qualitative factors that are not usually identified by the numerical (crisp) values. They are called fuzzy factors and can be explained by fuzzy antecedents such as ‘*my workload is very high and causes an extremely high work stress*’, ‘*the work-related stress is very low in my organization*’, ‘*managerial shortcomings are a highly effective source of work stress*’ etc.

**Figure 6:**
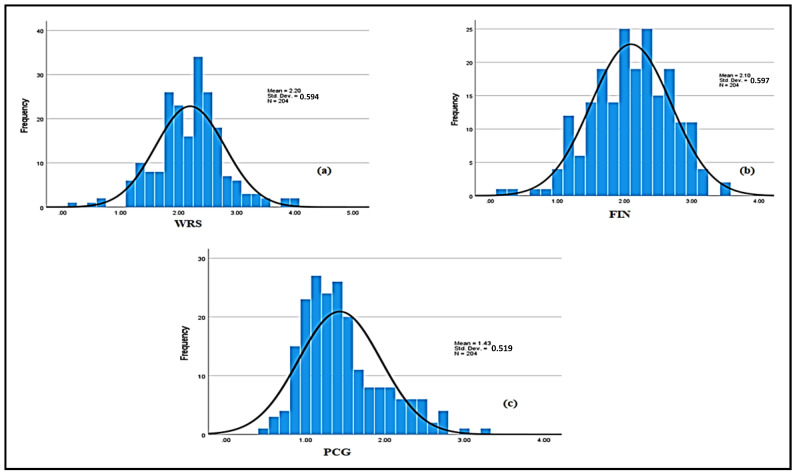
Normality plot of WRS (**a**), financial stress (**b**), and psychological stress (**c**).

Concurrently, a considerable number of staff indicated having varying degrees of WRS, ranging from ‘*a little bit*’ to ‘*moderate*’ due to different factors. The normal probability distribution shows that skewness coefficients of the data indicating the level of WRS and anxiety presented in Figure 6a, the financial factor presented in Figure 6b, and the psychological factor presented in Figure 6c, which came out to 0.0167, −0.2898, and 0.9295, respectively. Eventhough the data related to financial factors are very slightly skewed to the left, and the psychological factor is skewed to the right, the skewness coefficients fall within the range of normality. Likewise, the coefficients related to the kurtosis of WSR and anxiety, financial, and psychological factors are 1.253, −0.031, and 0.545, respectively, which depict normal distribution. For both indicators, the skewness and kurtosis signify that the data obtained conform to the normality criteria and can be utilized for further analysis. These numerical values just show a tendency of the dataset, however, and do not show how much the dataset is skewwed to the right or left. Therefore, WSR and anxiety, financial, and psychological factors can be better illustrated by fuzzy variables and their term sets.

#### 4.1.4. Socio-Demographic Factors (SCFs) vs. WRS and Anxiety

**Figure 7:**
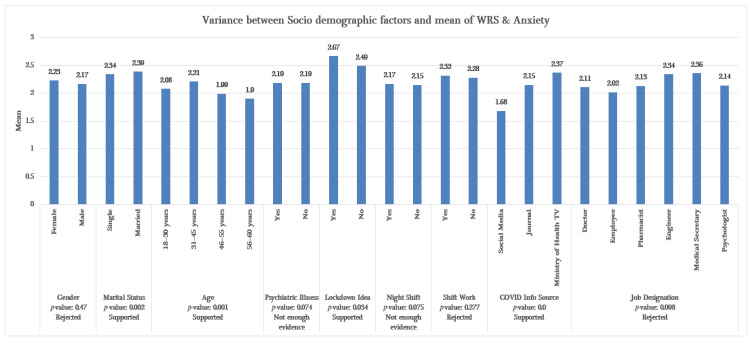
Variations among socio-demographic factors (SCFs) related to WRS and anxiety.

For the analysis of WRS and anxiety, an ANOVA test was also conducted for identifying socio-demographic factors. The outcomes indicate that marital status, age, lockdown, and the source of COVID-19 information significantly affected the variations in WRS and anxiety. A notable finding is that individuals who supported the effectiveness of lockdown measures tended to experience lower levels of WRS compared to those who held opposing views on lockdown measures. Younger staff reported a higher level of stress in contrast to older workers. Finally, the investigation revealed that individuals who relied on internet news coverage of COVID-19 tended to experience more stress than those who relied on journal reports [63] and were exposed to lower stress levels, as seen in Figure 7. Hence, the impact of psychological (PCG) and financial (FIN) factors on WRS and anxiety are presented in Table 1. The influence of the FIN factor was positively correlated with a WRS value of 0.401, indicating that higher financial strain was associated with increased WRS and anxiety. Likewise, psychological stress level showed a positive correlation of 0.376 with WRS and anxiety. In contrast, the number of staff residing with the other workers showed a negative correlation of −0.269 with WRS and anxiety. Also, time spent outdoors before lockdown showed a significant correlation with WRS and anxiety.

Although the influence of socio-demographic (SCF) factors cannot be presented by numerical values, the correlations between psychological (PCG) and financial (FIN) factors on WRS and anxiety are shown by numerical values in Table 1. Fuzzy logic can describe the relationships between numerical (crisp) and non-numerical (linguistic) variables in a much easier, more effective, and more understandable way. Fuzzy associations are the subjects of fuzzy logic and fuzzy inference systems to determine the relations of variables linguistically. Sugeno and/or Mamdani approaches [23,32,64] are usually used to reveal the relations of fuzzy linguistic variables, which will be clearly stated in Section 4.5.

Table 1 and Figure 7 helped us determine the strength of association of socio-demographic (SCF), psychological (PCG), and financial (FIN) factors with WRS and anxiety in generating fuzzy rules. Fuzzy association rule mining using fuzzy linguistic variables and their term sets forms the backbone of fuzzy logic and clearly reveals the relevant factors and their importance to assist domain experts. For example, there is a coefficient of 0.344 between the financial factor (FIN) and the psychological factor (PBF), which can be defined as ‘medium’ in terms of WRS and anxiety. Hence, these two linguistic variables are associated and have influence on work stress.

### 4.2. Fuzzy Clustering Analysis of WRS & Anxiety

**Figure 8:**
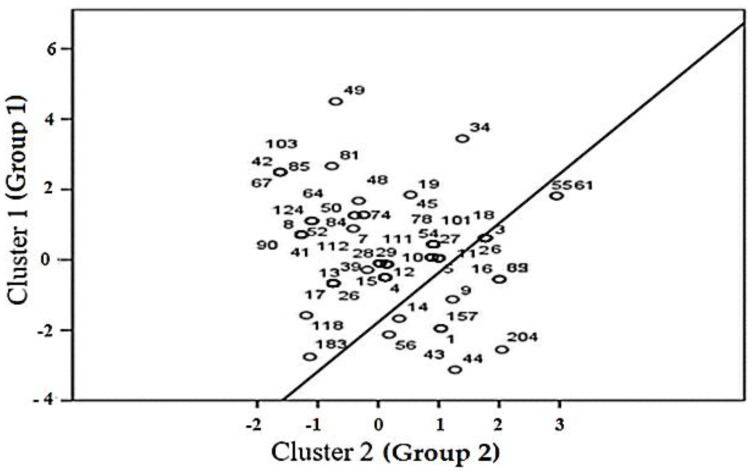
Distribution of clusters among demographic characteristics.

In this study, the K-means clustering algorithm was used to utilize the mean threshold to identify the center of a particular dataset. Hence, initially, two compatible groups were established from the dataset, as shown in Figure 8. Group # 1 comprised data of 175 staff workers who exhibit distinct characteristics positioned above the straight line, while group # 2 consisted of 30 data points positioned below the line. Thus, the data group indicates the respondents’ location and closeness to the other centers.

**Figure 9:**
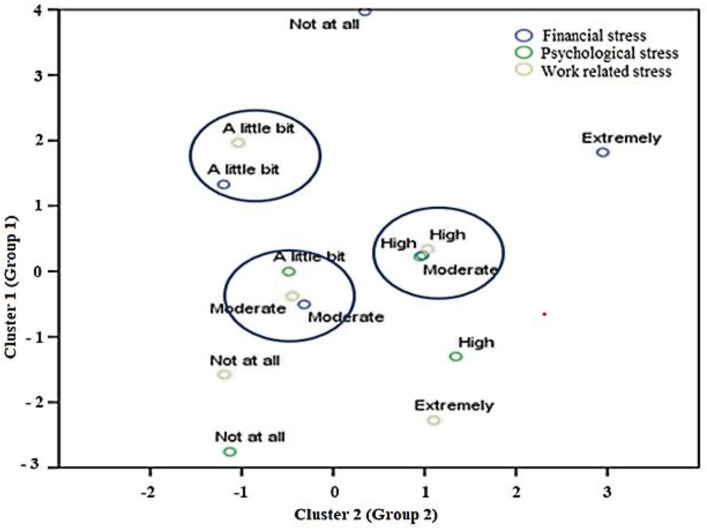
Cluster analysis of PCG, FIN, and WRS and anxiety used for fuzzy rule set development.

For instance, respondents 34, 49, 55, 61, and 183 are among those situated farthest within the group. In this study, the fuzzy clustering approach was used to generate objective number of groups (clusters) which could be used to depict the optimum number of fuzzy rules, the fuzziness level of clusters, and the number membership functions. Since the data in a cluster exhibit the same properties, each cluster can be represented by a membership function and a fuzzy rule. This approach raises a new debate and disadvantage, as a smaller cluster radius produces many data clusters and results in more association rules [59]. On the contrary, building clusters with large radii produces few fuzzy rules and is also far from achieving optimal number of rules (as seen in Figure 9). Therefore, a compromise is needed to optimize the cluster centers and the number of rules. Although many fuzzy association rules could be mined using this approach, our trial and the clustering approach helped determine that nine rules would be sufficient to associate the factors and predict the WRS and anxiety results.

In addition, chi-square and frequency analysis of the SCF factor within each cluster revealed the correlation of the clusters. Specifically, gender, age, diseases, lockdown, job designation, cohabitants, and time spent outside before lockdown were all associated with the clusters’ locations. However, patterns related to night shift workers were not statistically significant within the clusters. Hence, these people tended to consider lockdown as a beneficial decision, aligning with the characteristics of group # 1. In contrast, younger single survey participants tended to be more resistant to the idea of lockdown and relied more on the internet and social media news, have fewer partners, and spend more time outdoors. This group also displayed a higher likelihood of suffering from a diagnosis of a psychiatric disease.

The associations between the PCG, WRS and anxiety, FIN, and SCF factors suggest that individuals who were single or/and younger were diagnosed more with psychiatric disease. Staff who did not agree with lockdown, receive information from the media, work in the health sector administrative position, live with less than five people at home, and spend less time outdoors were more likely to experience higher levels of psychological stress. Similarly, single and younger staff in night shifts and/or day shifts who opposed lockdown, staff who gather information about COVID-19 from social media, and those who lived with few people at home were more likely to encounter higher WRS and anxiety.

In summary, the age, the number of cohabitants, and the source of COVID-19 information were found to be common factors affecting WRS and anxiety, PCG, and FIN stress factors.

### 4.3. Correspondance Analysis

The correspondence analysis illustrates the connection between the survey questions and factors. Particularly, PCG stress displayed less differentiation, suggesting that the stress levels are relatively closely situated. As depicted in Figure 9, WRS and anxiety showed the highest variation between cases, and this implies significant disparities between levels. In the meantime, financial stress (FIN) exhibited a remarkably high degree of variation at all levels apart from the staff who were in the ‘*moderate*’ category. As depicted in Figure 9, the staff in the ‘*not at all*’ and ‘*extremely*’ categories of stress are far different from the other levels. Staff who experienced ‘a little bit’ FIN stress also tended to report ‘a little bit’ WRS and anxiety. The WRS and anxiety level seems to be highly correlated with PCG and FIN stress; for instance, a staff worker who was ‘*moderately*’ under PCG and FIN stress also reported ‘*higher*’ WRS and anxiety. As a result, correspondence analysis helped identify term sets of fuzzy variables with hedges, which are special linguistic terms that are replaced by other linguistic terms such as a little, more, none, etc. Fuzzy association rules using fuzzy linguistic variables and their values form the backbone of fuzzy logic and clearly reveal the reletions of factors and their importance to assist domain experts for fuzzy rules formation. The performance of a fuzzy model depends entirely on two factors: the knowledge acquisition methods and the availability of domain experts used to transform human expertise into appropriate fuzzy ‘*If-Then*’ rules and the formation of membership functions (MFs) for a real-life problems. In addition, a rule base is the collection of domain knowledge acquired from a domain expert to show the relationship between inputs and output factors, eventually to develop a fuzzy association system. Also, fuzziness is a kind of imprecision stemming from grouping data that do not have sharply defined boundaries. In a nutshell, a sample fuzzy rule ‘*IF healthcare staffs are experiencing financial deterioration*, *THEN they may be likely to suffer psychological problems and change their socio-demographic location*’ is an association rule and can be mined from the dataset using simple fuzzy linguistic terms.

### 4.4. Structural Equation Modeling (SEM) Approach

Punniyamoorthy et al. (2010) [65] used the SEM approach to indicate the relationship between input factors and outcomes to arrive at the relative weight of the factors and to select the right metrics. Hafish et al. (2024) [66] used SEM and fuzzy set comparative qualitative analysis with 206 samples from Indonesia to model and examine green procurement adoption. In an SEM approach, chi-square distribution/degrees of freedom (CMIN/DF ($\chi^{2}/df$)) and root mean square error (RMSE) metrics were employed to measure the absolute goodness of fit [67]. Similarly, the incremental goodness of fit was determined using a comparative fit index (CFI), yet the absolute goodness of fit was measured by the goodness-of-fit index (GFI) [63]. As shown in Table 2, the model fit measurements CMIN/DF, RMSE, and ‘*p*’ values were calculated as 2.78, 0.09, and 0.00, respectively. As a result, it can be said that the model fits well.

Thus, in the initial model of our study, survey questions such as staff workload, poor management, lack of support, poor relationships, infection stress, access to morning sunlight, and meeting urgent needs (WRS-1, WRS-2, WRS-3, WRS-4, PCG-2, PCG-13, and FIN-5) exhibited extremely low weight factor loadings. Therefore, these factors were not considered in the rule-based fuzzy association mining process. For instance, as detailed in Table 3, factors such as financial effects and debt payment challenges (FIN-3 and FIN-6) had the most substantial influence, accounting for over 0.85 and 0.63, respectively, of the financial stress experienced by health workers in Saudi Arabia. Among the 15 subclusters, including PCG factors, PCG-10 and PCG-11 were found to be the source of depression, pessimism, occasional crying, and loss of motivation, which exhibited effects surpassing 0.850 and 0.810, respectively. In the context of WRS and anxiety, WRS-5 and WRS-6, pertaining to technology and teamwork, showed a 0.697 and 0.745 impact, respectively, on WRS and anxiety. Thus, we took these factors into account when developing the FARM process and for establishing the fuzzy rules. Conversely, while the SEM indicated that FIN stress was not significantly effective among healthcare staff in Saudi Arabia, it is hypothesized that the complex internal effects and correlations within the SEM lead to important consequences for staff motivation; thus, this factor was included in the development of the fuzzy rule base. In the meantime, the correlations between SCF impact, TECH impact, FIN, and PCG impact are noteworthy for their positive, negative, and substantial nature and nine fuzzy rules-based associations.

### 4.5. Application of FARM and Fuzzy Transactions to WRS and Anxiety

Data mining is an Artificial Intelligence (AI) technique employed for the detection of knowledge in large datasets and extract hidden information to uncover real-life problems in many fields [67]. In this study, MATLAB R2021a, IBM SPSS v18.5 Modeler’s data mining and text analytics software, statistical algorithms, and data mining algorithms (Apriori algorithm) were used to analyze the collected survey data for developing a predictive AI model. The FARM approach-related rules were derived from the statistical analysis presented in Section 4.1, Section 4.2, Section 4.3 and Section 4.4. Additionally, Section 3.2 is about the theoretical background of fuzzy association rules mining (FARM) approach and fuzzy transactions suggested in our work. The application of FARM is presented in Section 4.5 with all its dimensions and details. Figure 1 shows the step-by-step detail of the FARM approach for WRS and anxiety prediction.

For instance, the statistical analysis presented in Section 4.1 was used for analyzing the collected data, and the quantitative and qualitative data were identified to accurately determine the fuzzy linguistic variables and their term sets. In addition, Table 1 was used to identify the correlation between the psychological and financial factors and WRS and anxiety for clarifying the impacts of these predictors and for developing fuzzy rule sets. Moreover, the ANOVA conducted clearly outlined the relevant factors and their significance to help domain experts establish fuzzy rules and identify fuzzy linguistic factors.

Our analysis and the judgments of domain experts shows that the fuzzy variables and term sets presented in Table 4 can be used for FARM model development and for the prediction of WRS and anxiety. After determining the fuzzy variables and their term sets, the next step was to determine fuzzy rule base for the FARM model development. Therefore, in Section 4.2, the fuzzy clustering approach is presented to generate the objective number of data groups (clusters) for determining the optimum number of fuzzy rules, the fuzziness level of clusters, and the membership functions. In this work, the numbers of rules were generally considered equal to the number of clusters. In addition, the K-means clustering algorithm was used to benefit from cluster centers and determine the optimum number of fuzzy rules. After extensive analysis of the survey data, nine near-optimal clusters were found to form the fuzzy association rule mining approach, as seen in Figure 8 and Figure 9.

In addition, Section 4.3 detailed the correspondence analysis to illustrate the connection between the survey questions and fuzzy variables. This analysis also served to establish fuzzy association rules. Fuzzy association rules are ‘*If-Then*’ structures to connect the fuzzy linguistic factors and their terms, which form the backbone of fuzzy logic and FIS.

**Figure 10:**
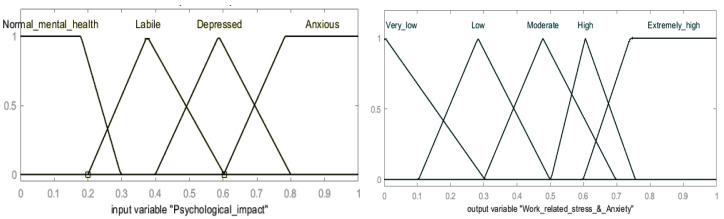
Membership functions of fuzzy variables for association rule mining.

The prediction performance of an FIS model depends entirely on accurately transforming human expertise into appropriate fuzzy rules and membership functions (MFs). This depends on how knowledge engineers transform qualitative and quantitative data into fuzzy rules. In fact, there is not a single way or limit to the information transformation that AI systems can recognize. Several techniques are available like fuzzy logic, fuzzy sets and systems, reasoning, etc. However, highly accurate knowledge of a topic will be very useful in producing meaningful AI models. Figure 10 illustrates the triangular MFs employed in this study. MFs are the mathematical tools used to present the fuzzy or crsip values of a variable in terms of membership degrees and fuzzified levels. They are the only way to identify the linguistic statements in fuzzy mathematics to solve vagueness and imprecision problems. The fuzzy linguistic variables and terms presented in Table 4 were used for the MFs and their fuzziness level and intervals. The vagueness and imprecision of fuzzy terms are termed as fuzziness and stem from grouping data that do not have sharp and clear boundaries.

Section 4.4 gives the details of the SEM approach which was used to determine the weights of fuzzy factors considered in the FARM process. The numerical (crisps) weights of health factors have been presented in Table 3 to identify the direct and indirect effects of the factors on WRS and anxiety. For instance, although FIN stress appeared not to be significantly effective among healthcare staff in Saudi Arabia, this factor had very important consequences on the motivation of the staff and therefore was considered for the fuzzy rule base development in our work. Additionally, SEM helped us analyze the technological impact (TECH) and its positive and negative effects in fuzzy rule-based attribution.

**Figure 11:**
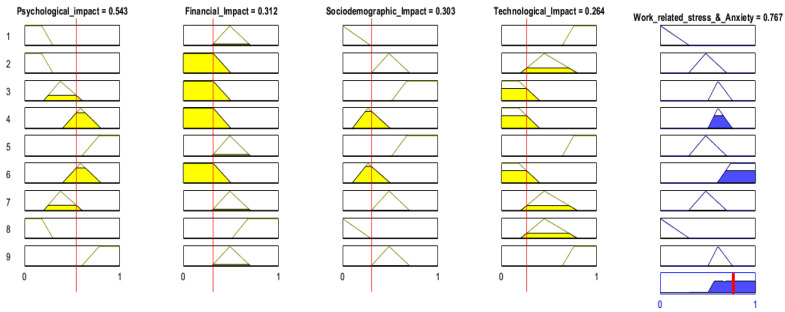
Fuzzy inference system for association rules mining of WRS and anxiety.

In a nutshell, nine rules (see in Figure 11) were constructed for the FARM approach, and four of them were presented in Table 5. A sample fuzzy rule can be presented as ‘*IF healthcare staff are experiencing financial deterioration*, *THEN they may be likely to suffer psychological problems and change their socio-demographic location*’ to identify the association rule using fuzzy linguistic factors and terms. The FARM approach consists of combining rule mining to identify relationships and frequent patterns among a set of elements in a database [68]. In association rules mining (ARM), the importance of a rule is usually measured by criteria sets.

As are shown in Figure 8 and Figure 9, fuzzy partitions were specified within the domain of data obtained from a survey of healthcare staff; thereby, the dataset was transformed into attribute values within the interval [0, 1] for the establishment of the FARM process. The development of FARM is directly attributed to the fuzzy linguistic variables, and their term set presented in Table 4, while the membership functions given in Figure 10. FARM is a rule mining approach used to identify operations of linguistic variables and terms to establish rules using natural language. The FARM approach can produce more dependable and accurate solutions even if the health data overlap and are positioned in neighboring boundaries. This is the advantage of using fuzzy logic in cases where there are not clear cuts of boundaries of variables. FARM uses fuzzy set theory considering linguistic variables for building explainable reasoning systems and is a well-known data mining method to identify repeatedly occurring patterns from datasets. In the case where the relations of factors are not clear, for uncovering hidden relationships between the factors or identifying the overlapping data and interpreting them, Boolean association rules are not suitable. However, the fuzzy inference system (FIS) uses fuzzy logic to clarify uncertain and imprecise relationships by using fuzzy linguistic variables and their linguistic term sets, membership functions, a fuzzy rule base, a fuzzification process, an inference engine, and defuzzification.

It should be noted that a disease related to WRS and anxiety may emerge from more than one source; therefore, the factors’ boundaries are unclear and vague. For instance, the reason for a psychological disease may be due to financial reasons or bad management in the workplace. However, statistical approaches cannot analyze the mutual multifactorial effects of health problems on WRS and anxiety, which can only show their effects in a narrow area.

On the contrary, fuzzy extension of the ARM method can contribute to closing this gap. The value of fuzzy support (F-supp) calculated by Equation (3) shows the confidence threshold value of each rule; all possible fuzzy association rules can be extracted in this way. We have calculated the fuzzy support value of the top nine fuzzy association rules and the corresponding confidence values. The fuzzy support and confidence values of the rules are given in Table 6. The proposed FARM approach can consider different rates of WRS and anxiety, whereas the traditional ARM approach only considers binary scales. The fuzzy variables and terms presented in Table 4 are unique and were determined only for this study. The triangular fuzzy MFs given in Figure 10 were determined by healthcare domain experts, considering all the findings and similar issues presented in Section 4.1.

The Mamdani fuzzy inference system (FIS) presented in Figure 11 has nine rules for the rule-based system. The Mamdani approach is a well-known FIS model, which was first introduced to create a control system by combining fuzzy linguistic rules obtained from experienced domain experts. Since Mamdani FISs are more intuitive rule-based systems, fuzzy rules are created from the knowledge of domain experts. The advantages of the Mamdani FIS are that it is intuitive, adapts well to human inputs, has a more interpretable rule base, and is widely accepted.

Figure 11 shows that rule #4 and rule #6 were fired, and the medical staff who were in this class had moderately high WRS and anxiety levels. This is because if this staff group’s ‘*psychological impact is in the depressive mode (0.534) and financial impact is deteriorating (0.312) and socio-demographic impact is mildly associated with anxiety (0.303) and is technologically negatively associated with stress (0.264)*’ *then he/she falls into the category that WRS and anxiety are moderately high (0.767)*’. Yet, 0.767 is the total fuzzy membership degree and was calculated using the fuzzy aggregation operator. As given in Table 6, the fuzzy support and confidence values of rule # 4 are 0.56 and 0.57, respectively, which also prove that this rule is significant enough to be used for the FARM model.

Several fuzzy association rules can be mined using this approach; hence, WRS and anxiety can be predicted from these mined rules and the effective parameters can be determined by the FIS given in Figure 11. Applying the Mamdani FIS, for the prediction of WRS and anxiety, not necessarily all rules have to be fired, usually one rule or sometimes many rules are fired together to predict the outcomes. If multiple rules are fired, for obtaining the outcomes, compositional rules of inference approach are employed. The fuzzy support and confidence values of the nine rules were calculated and are presented in Table 6, and then the Apriori algorithm was run for all combinations of frequencies of WRS and anxiety for medical staff. Additionally, the significance of the rules was determined by statistical chi-square test and fuzzy support, and the confidence values of the rules were calculated to eliminate redundant rules and determine the most significant rules.

According to the results and findings obtained, the total fuzzy score of staff #9 is 0.916 (as seen in Table 7) and is larger than the minimum fuzzy support value of 0.36 (as seen in Table 6). The most effective criteria for this staff is the ‘*psychological impact*’; its rate is 0.959, and this staff is considered in the ‘anxious mood’ and his/her WRS and anxiety is extremely high. Additionally, his/her financial impact (FIN) ratio is 0.635 (improved), the socio-demographic impact (SCF) ratio is 0.936 (strong association), and the technological impact (TECH) ratio is 0.875 (positively impact improve stress in workplace). The psychological impact (PCG) ratio and the socio-demographic impact (SCF) ratio are the most influential factors for this staff. Hence, using the knowledge in Table 7 and FIS, the total fuzzy score of WRS and anxiety of any medical staff can be determined.

Similarly, the total fuzzy score of staff #3 is 0.529, which is higher than the minimum fuzzy support value of 0.45. The most effective criterion for this staff is the ‘*Technological impact*’, its rate is 0.716, and this staff is considered in the ‘*moderate*’ mood; additionally, his/her ‘*psychological impact*’ ratio is 0.628, financial impact (FIN) ratio is 0.402, and socio-demographic impact (SCF) ratio is 0.564.

**Figure 12:**
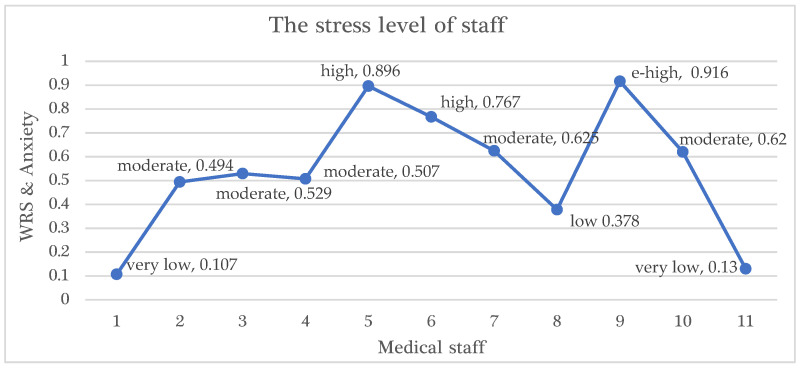
The stress level of staff measured by FARM approach.

FARM, developed for the validity of FIS, was used to estimate the WRS and anxiety of 11 staff, and the findings and results are presented in Table 7. In addition, the stress level of these 11 staff is presented in terms of fuzzy numerical values and their linguistic terms in Figure 12.

Clearly, the FARM approach can predict the WRS and anxiety level of any staff. In a nutshell, it should be stated that the improvement in the financial status of people was not sufficient alone for the healthcare staff during the pandemic. Although psychological impact alone is a very important and significant variable in WRS and anxiety assessment, along with the financial impact, it has greater impact on staff’s emotion and WRS and anxiety. The results and findings also depicted that when the financial burden (FIN) and psychological stress (PCG) of healthcare staff increased, WRS and anxiety increased. This is due to the mutual effects of these two factors on the staff’s motivation, which can only be determined by the FARM approach.

## 5. Conclusions and Discussions

While WRS and anxiety were not considered a serious problem in the early stages of the COVID-19 pandemic, in the later period, mass deaths were encountered in many countries when its scope and effects emerged and were understood in some countries in Europe and other places. Therefore, the contribution of healthcare staff became much more important and inevitable at that time. It was also important to examine the factors that caused stress and anxiety in healthcare workers, the interactions of the factors with each other, and their effects. In addition, the motivation of staff was crucially important to provide better and higher quality service to the patients during pandemics in the hospitals, because the healthcare sector requires high staff motivation, low WRS and anxiety, and a productive working environment. For instance, Zare et al. (2021) [69] assessed the occupational stress of health care workers in Kerman province hospitals in Iran, where they performed an analytical study on 290 medical staff workers, including nurses, physicians, and cleaning crew working with COVID-19 patients. Although most of their factors such as communications, manager support, and change could not be explained well by the numerical values, they employed traditional statistical approaches for the assessment and analysis of the problem. Similarly, Lancet (2020) [70] studied the COVID-19 pandemic for protecting healthcare workers using traditional approaches. The findings were not as abvious as the findings of our work. Hence, fuzzy logic and fuzzy sets and systems can contribute to these types of work and help explain the value of factors in a much clearer way.

In this study, a survey was conducted among the healthcare sector employees working in public and private organizations to obtain qualitative and quantitative data in Saudi Arabia. The dataset was analysed by several techniques such as correspondence analysis, the structural equation model, and the fuzzy association rule mining (FARM) approach to analyze the WRS and anxiety of healthcare staff. The results and outcomes of correspondence analysis, structural equation modeling, and clustering analysis were employed for the development of a FARM approach for obtaining and analyzing WRS and anxiety levels. The developed FARM is a data mining technique and works based on fuzzy rules to obtain the WRS and anxiety measures of healthcare staff. The qualitative metrics and quantitative data analysis of statistical methods were employed to develop the FARM approach. For instance, the correspondence analysis revealed the connection between survey questions and factors. For example, the lower variability in psychological stress suggests that the stress levels of employees located close to each other are more dependent on other factors. As a result of the analysis and synthesis, WRS and anxiety showed high variability among cases (staff), and significant different stress levels were observed due to the different expectations, unknown characteristics of pandemic, and different nationalities of the staff, as seen in Table 7.

The structural equation model (SEM) determined the standardized weights of each latent effective factor to its corresponding measured variable. SEM exposed the correlation between demographics characteristics of staff and both financial and psychological stress of them, hence achieving remarkable conclusions. For example, SEM depicted that financial stress was not significantly influential among healthcare staff in Saudi Arabia but showed a remarkable high variability among them. However, it was considered in the study due to its significant internal effects and its very significant consequences on the motivation of the staff.

The standardized direct and indirect total effects of the parameters and their consequences on WRS and anxiety were also determined by this approach. For instance, psychological factors, (PCG-10 and PCG-11) were found to be the causes of depression, pessimism, occasional crying, and loss of motivation in this work. Additionally, the fuzzy impacts of technology and teamwork were found to be 0.697 and 0.745 on WRS and anxiety, respectively. Conversely, the utilization of technology within the hospital was not identified as a stress-inducing factor. More than 70% of healthcare specialists affirmed that teamwork did not contribute to the WRS negatively. Lastly, the investigation revealed that individuals who relied on internet news coverage of COVID-19 tended to experience more stress than those who relied on journal reports and were exposed to lower stress levels. Younger staff reported a higher level of stress in contrast to older workers.

FARM uses fuzzy set theory and is a well-known data mining method to identify recurrently occurring patterns. The developed FIS uses fuzzy logic to clarify vague and imprecise relations via fuzzy linguistic variables and their term sets, membership functions, a fuzzy rule base, a fuzzification process, an inference engine, and defuzzification. Therefore, it can be clearly stated that by using the FARM approach, the fuzzy associations and the most effective factors were determined for predicting the WRS and anxiety of 11 healthcare staff presented in Table 7. As a result, it can be said that the FARM model can predict WRS and anxiety and determine the effectiveness of key factors. For example, if a staff member is psychologically in normal mental health (0.239), financially is in a normal condition (0.637), has a positive socio-demographic impact (0.168), and their hospital is technologically in a good condition (0.644), then the staff’s WRS and anxiety value is very low (0.12).

The limitations of this study can be as follows. Knowledge acquisition is a kind of bottleneck in AI-based FARM approach development. On the other hand, validation and formal difficulties have arisen in the acquisition of medical information, especially due to ethical issues regarding the rights of healthcare staff. FARM can be used in a non-invasive way for solving social problems and helps us scientifically or medically relate the causes and effects between the observed phenomena. As a further study, the size of the model can be extended by taking more health parameters into account. Hence, taking various factors into account and more numbers of participants can improve the accuracy of findings and generalize the results and outcomes.

Furthermore, healthcare personnel from many hospital departments participated in the survey, with no goal of picking a certain kind of staff for the study. This condition may restrict the generalizability of the findings to other healthcare personnel due to the disparity in the numbers of doctors and nurses. Consequently, significant challenges emerge, particularly for medical knowledge bases, due to their amalgamation of inaccurate and ambiguous information, which could lead to potentially life-threatening outcomes if therapies are administered incorrectly. Nevertheless, we neither possess nor utilize this type of knowledge in our endeavors.

As a conclusion, it appears that factors such as high workload, insufficient managerial support to the critical cases and situations, lack of adequate techical protective equipment, socio-demographic changes, and financial problems influenced the stress levels among the healthcare staff. Due to the possibility of recurrence of COVID-19-like pandemics, the importance and involvement of healthcare staff, preparation of hospital conditions, as well as responsiveness to emergency cases are crucial requirements for any nation. Thus, the findings of this study may serve as a reference for subsequent actions, including the execution of interventions during the pandemic to mitigate occupational stress, sustain job stability, and enhance the quality of life of healthcare staff.

## Figures and Tables

**Table 1:** Correlations between psychological and financial factors of WRS and anxiety.

Correlation	Psychological Factor (PCGs)	Financial Factors (FINs)	WRS and Anxiety	People Living Together	Time Spent Outdoors Before Lockdown
Psychological factors (PCGs)	1				
Financial factors (FINs)	0.344	1			
WRS and anxiety	0.376	0.401	1		
People living together	−0.210	0.337	−0.269	1	
Times spent outdoors before lockdown	0.255	0.270	0.425	−0.172	1

**Table 2:** The results and findings of structural equation model.

Model Fitness Index	$\chi^{2}/df$	GFI	CFI	RMSE	*p*-Value
Model value	2.78	0.82	0.75	0.09	0.00
Recommended value	<5	>0.9	>0.9	<0.09	<0.05

**Table 3:** Total direct and indirect effects of the factors on WRS and anxiety.

Sources of Occupational Stress	Financial Stress	Psychological Stress	Total WRS and Anxiety
WRS-5	0.142	0.463	0.697
WRS-6	0.152	0.495	0.745
FIN-6	0.630	0.000	0.000
FIN-4	0.412	0.000	0.000
FIN-3	0.850	0.000	0.000
FIN-2	0.466	0.000	0.000
FIN-1	0.173	0.000	0.000
PCG-1	0.000	0.590	0.000
PCG-3	0.000	0.632	0.000
PCG-4	0.000	0.393	0.000
PCG-5	0.000	0.628	0.000
PCG-6	0.000	0.482	0.000
PCG-7	0.000	0.350	0.000
PCG-8	0.000	0.606	0.000
PCG-9	0.000	0.464	0.000
PCG-10	0.000	0.848	0.000
PCG-11	0.000	0.810	0.000
PCG-12	0.000	0.390	0.000
PCG-14	0.000	0.238	0.000
PCG-15	0.000	0.346	0.000

**Table 4:** Fuzzy variables and term sets for WRS and anxiety.

Fuzzy Variables	Fuzzy Term Sets
Psychological (PCG) impact	normal mental health, labile (mood swing), depressed, anxious
Financial (FIN) impact	deteriorating, not changed, improved
Socio-demographic (SCF) impact	not associated, mild association, moderate association, strong association,
Technological (TECH) impact	negative impact, no impact, positive impact improve stress in workplace
Work-related stress and anxiety	very low, low, moderate, high, extremely high,

**Table 5:** Fuzzy association rules with fuzzy confidence values.

	Fuzzy Rules	Fuzzy Confidence
***Rule 1***	If (Psychological_impact is depressed) and (Financial_impact is deteriorating) and (Sociodemographic_impact is mild_associated) and (Technological _impact is negative) then (WRS and Anxiety is moderate high (0.767).	0.76
***Rule 2***	If (Psychological_impact is labile) and (Financial_impact is not_changing) and (Sociodemographic_impact is moderately_associated) and (Technological_impact is positive) then (WRS and Anxiety is moderate (0.496).	0.92
***Rule 3***	If (Psychological_impact is in normal mental_health) and (Financial_impact is not changing) and (Sociodemographic_impact is not_associated) and (Technological _impact is High) then (WRS and Anxiety is very_low) (0.13).	0.71
***Rule 4***	If (Psychological_impact is depressed) and (Financial_impact is deteriorating) and (Sociodemographic_impact is Strong_association) and (Technological _impact is Negative_impact) then (WRS_and_Anxiety is extremely_high) (1).	0.57

**Table 6:** Fuzzy rules and their support and confidence values.

Fuzzy Rules	Fuzzy Support	Fuzzy Confidence
1	0.36	0.76
2	0.39	0.92
3	0.45	0.71
4	0.56	0.57
5	0.62	0.35
6	0.72	0.63
7	0.41	0.53
8	0.52	0.56
9	0.51	0.67

**Table 7:** The stress level identification and the impact of factors on medical staff’s WRS and anxiety.

Medical Staff	Fuzzy Input Parameters				The Stress Level of Staff	Stress Level in Terms
	PCG Impact	FIN Impact	SCF Impact	TECH Impact	WRS and Anxiety	WRS and Anxiety
1	0.184	0.5	0.0947	0.731	0.107	very low
2	0.244	0.387	0.428	0.633	0.494	moderate
3	0.628	0.402	0.564	0.716	0.529	moderate
4	0.718	0.538	0.663	0.716	0.507	moderate
5	0.898	0.643	0.761	0.867	0.896	high
6	0.534	0.312	0.303	0.264	0.767	high
7	0.695	0.620	0.375	0.913	0.625	moderate
8	0.237	0.199	0.337	0.223	0.378	low
9	0.959	0.635	0.936	0.875	0.916	e-high
10	0.750	0.519	0.511	0.772	0.620	moderate
11	0.146	0.332	0.153	0.690	0.130	very low

## Data Availability

The data presented in this study are available upon request from the corresponding author due to regulations from the research ethics committee at King Abdulaziz University.
